# Depression, Anxiety, and Stress Symptoms in Patients With Beta Thalassemia Major in Almadinah Almunawwarah, Saudi Arabia

**DOI:** 10.7759/cureus.11367

**Published:** 2020-11-07

**Authors:** Mohammed A Zolaly, Farah M Zolaly, Lama Al Belowi, Raafat Shuqdar, Mohammed A Al Belowi, Turki A Alwasaidi, Muayad Albadrani

**Affiliations:** 1 Hematology and Oncology, College of Medicine, Taibah University, Madinah, SAU; 2 Internal Medicine, College of Medicine, Taibah University, Madinah, SAU; 3 Psychiatry, College of Medicine, Taibah University, Madinah, SAU; 4 Hematology, College of Medicine, Taibah University, Madinah, SAU; 5 Family and Community Medicine, College of Medicine, Taibah University, Madinah, SAU

**Keywords:** anxiety, chronic disease, depression, saudi arabia, stress, thalassemia

## Abstract

Introduction

Beta thalassemia major (BTM) is a chronic hereditary blood disorder. Patients are dependent on blood transfusion and are prone to multiple comorbidities. Depression, anxiety, and stress (DAS) can complicate their condition. No reports from Saudi Arabia to measure DAS in BTM patients. We report the prevalence of DAS symptoms in our BTM patients using the DASS-21 (Depression, Anxiety, and Stress Scale - 21 Items) test.

Methods

A cross-sectional study including adolescents and adults aged 14 years and above with BTM treated in Almadinah Almunawwarah and excluding patients who had bone marrow transplant or with central nervous system insults were performed.

Results

A total of 31 male and 31 female patients were enrolled, with a mean age of 24.32 ± 7.05 years. Depression symptoms were detected in 60 % of patients, anxiety symptoms were detected in half of the studied group, and stress symptoms were detected in 38.7% of patients. We found a significant positive correlation between DAS and DASS total score. Age below or above 18, parent’s employment, patient’s educational level, and status of satisfaction about medical care were statistically significant in having positive effects on scores of DAS symptoms.

Conclusions

BTM patients are prone to develop psychological disorders, which can affect the course of the disease. Our results are comparable to international and Arab population studies, which have the highest reported prevalence. It is important to not ignore the psychological evaluation of patients with BTM and to refer them for proper evaluation and management.

## Introduction

Beta thalassemia major (BTM) is a chronic blood disorder caused by inheritance of two abnormal genes in an autosomal recessive pattern. It is usually diagnosed in early childhood as patients present with anemia and signs of hemolysis [1,2]. Patients with BTM are dependent on blood transfusion for life, unless they undergo bone marrow transplant. Frequently, they are prone to develop multiple complications from their disease and from the treatment they are receiving [3,4].

Depression, anxiety, and stress (DAS) are psychological disorders, which can complicate BTM patients’ condition and cause multiple comorbidities if they were not diagnosed early and managed appropriately [5-8].

It is expected and well documented in the literature that the diagnosis of BTM patients with DAS can affect the course of the thalassemia disease and if detected early and managed appropriately will improve the outcome including compliance with treatment and hence improve the quality of life of affected patients [7].

Thalassemia is a chronic disease with a lot of psychological impact on patients, such as coming to the hospital every month and being afraid of blood-transmitted infections, having to take iron chelators every day, getting complications of iron overload, missing school and work to attend the blood transfusion sessions, financial burden, loss of friends and family members from the disease, hormonal abnormalities, and disfiguring abnormalities from bone marrow expansion [5-8].

A very serious complication of repeated blood transfusion is iron overload and deposition of iron in different body organs for which iron chelators are prescribed to all suffering patients. Issues with poor compliance can be linked to loss of hope and low self-esteem in those patients [9].

Involving psychiatric services and psychological support is part of the multidisciplinary approach that patients with thalassemia need so as to ensure an effective comprehensive care that they deserve, but unfortunately this is not always utilized [8].

The Depression, Anxiety, and Stress 21-Item Scale (DASS-21) is a screening tool for DAS, which was validated and recently translated to Arabic language and proved effective in an Arabic-speaking population, which is a shorter form of DASS-42 questionnaire, which was applied and tested in different parts of the world and therefore was chosen as a tool for reporting the prevalence of depression, anxiety, and psychological stress in our BTM patients [10,11].

To date, we are not aware of any reports from Saudi Arabia that measures DAS in BTM patients, and therefore our aim in this study is to screen our BTM patients in Almadinah Almunawwarah for these symptoms with the hope of detecting high-risk patients and refer them for further appropriate evaluation and management.

Almadinah Almunawwarah region is located on the western side of Saudi Arabia. Diversity of ethnicity is unique among the population of Almadinah Almunawwarah, and the latest population reported is 1.2 million [12].

Two large hematology centers located in both Madinah Maternity and Children Hospital (MMCH) and King Fahad Hospital (KFH) in Almadinah Almunawwarah serve more than 3,000 patients with hematological disorders. Thalassemia patients are followed in one of these two centers for more than 30 years now, and a total of 111 patients with thalassemia major are currently receiving regular blood transfusion in one of the two centers and are being followed up regularly.

## Materials and methods

This is a cross-sectional questionnaire-based study conducted using telephonic interviews with patients.

The inclusion criteria for the study included adolescents and adults aged 14 years and above diagnosed with BTM who are transfusion-dependent and treated in MMCH and KFH in Almadinah Almunawwarah.

We excluded patients who had bone marrow transplant and were cured of thalassemia as well as patients with central nervous system insults, such as stroke, preventing them from expressing themselves and not able to communicate well.

The questionnaire includes demographic data including age, gender, nationality, order in their family, income, education, hospital followed up at, presence of disease in other members of the family, and if they have a history of deaths related to thalassemia diagnosis in their family.

Disease history information included frequency of blood transfusion, hemoglobin level before blood transfusion, serum ferritin levels, history of surgical splenectomy, and iron chelators usage. We also screened patients for the ongoing support they were getting, including support from family and the medical team, and the evaluation of level of satisfaction from medical care and psychological care that had been received.

DASS-21 screening questions consist of 21 questions to evaluate depression (seven questions), anxiety (seven questions), and stress (seven questions).

Ethical approval was obtained from the Research Ethics Committee of Taibah University. A consent was obtained from all participants who agreed to join the study after explaining the purposes. All data were dealt with high confidentiality standards and kept in a safe place. The questionnaire was initially piloted on 10 patients and was proven effective and easy to apply.

The statistical analysis in our study was carried out using SPSS Version 27 for Windows (IBM Corp., Armonk, NY, USA). The level of significance was set at 5%. The characteristics of the sample were summarized using means and standard deviations (SDs) for continuous variables and as frequencies with their percentages for categorical variables. An independent samples t-test was used for continuous variables in the comparison of two groups’ means, whereas analysis of variance (ANOVA) was used to compare the differences in scores of the variables of more than two groups.

## Results

**Figure 1:**
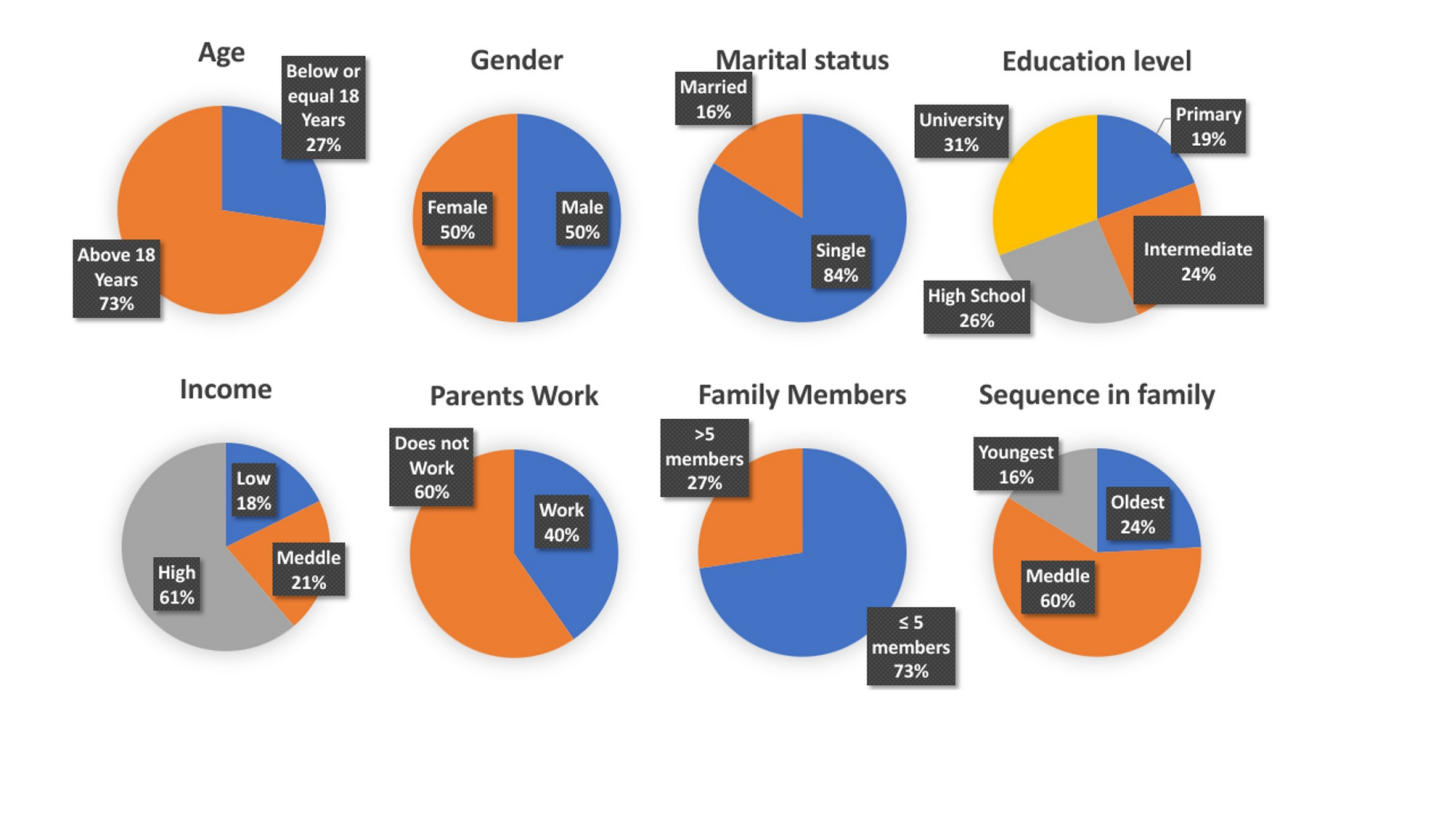
Epidemiological data

The analysis was performed on 62 patients with thalassemia who joined the study. In Figure 1, the sociodemographic characteristics of the participants are shown. Their mean age was 24.32 ± 7.05 years: 17 (27.4%) participants were 14-18 years old and 45 (27.6 %) were ≥ 19 years old. Of the patients, 31 were males, whereas 31 were females. In terms of educational level, 19.4%, 24.2%, 30.6%, and 25.8% of the participants completed the primary, intermediate, high school, and university levels, respectively.

In terms of employment of the participants’ parents, 60% were unemployed. Of the participants, 72.6% had a family with five or fewer members, whereas 24.7% had more than five members. As for birth order, the majority (59.7%) participants were middle born, 24.2% were the eldest, and 16.7% were the youngest in the family. Furthermore, 71% had at least one family member affected with the same illness, whereas 29% of them were the only ones having BTM in the family.

The participants’ categories in terms of the severity of DAS symptoms based on their DASS 21 scores are shown in Table 1. The mean depression score of the study population was 5.45 (SD = 4.09), ranging from 0 to 14, of which 11% (7) of the participating patients were within the severe to extremely severe range of stress, 29% (18) were within the moderate category, and 21% (13) were categorized as mild. The remaining participants (38%; 24) were categorized as normal.

**Table 1:** Severity of depression, anxiety, and stress symptoms based on DASS-21 score among the participants. DASS-21, Depression, Anxiety and Stress Scale - 21 Items

Symptoms profile based on DASS-21 score		Frequency	%
Stress	Normal (0-7)	38	61.3
	Mild (8-9)	8	12.9
	Moderate (10-12)	10	16.1
	Severe (13-16)	5	8.1
	Extremely severe (17+)	1	1.6
Anxiety	Normal (0-3)	31	50
	Mild (4-5)	13	21
	Moderate (6-7)	4	6.5
	Severe (8-9)	7	11.3
	Extremely severe (10+)	7	11.3
Depression	Normal (0-4)	24	38.7
	Mild (5-6)	13	21
	Moderate (7-10)	18	29
	Severe (11-13)	5	8.1
	Extremely severe (14+)	2	3.2

The mean anxiety score of the study population was 4.37 (SD = 3.06), ranging from 0 to 14. We discovered that 23% (14) of the participating patients were within the severe to extremely severe range of anxiety, 6.5% (4) were within the moderate range of anxiety, and 21% (13) were categorized as mild, whereas half of the participants (50%; 31) were categorized as normal.

The mean stress score of the study population was 6.37 (SD = 4.35), ranging from 0 to 17. It was found that 9.7% (6) of the participating patients were within the severe to extremely severe range of stress, 16.1% (10) were within the moderate category, and 12.9% (8) were categorized as mild, whereas the rest of the participants (61.3%; 38) were categorized as normal.

We found that there is a significant correlation between depression, anxiety, stress, and DASS total score based on Pearson’s correlation coefficient, as shown in Table 2.

**Table 2:** Correlations between DASS-21 total score and other scores. DASS-21, Depression, Anxiety and Stress Scale - 21 Items *p < 0.1

Variable	DASS-21 total	Stress score	Anxiety score	Depression score
DASS-21 total	1	0.884*	0.842*	0.849*
Stress score	0.884*	1	0.612*	0.621*
Anxiety score	0.824*	0.612*	1	0.540*
Depression score	0.849*	0.621*	0.540*	1

The results of independent samples t-test analysis and one-way ANOVA between DAS and the demographic variables are presented in Table 3. The group with age ≤18 years had lower mean scores for DAS. The difference in average stress scores between the group with age ≤18 years and the group with age of more than 18 years was statistically significant (p = 0.033). The difference in average depression and anxiety scores between the different age groups was not statistically significant but higher in the older group. Males showed higher mean scores for depression and stress than females. Married participants obtained higher mean scores for DAS than single participants did. Participants with a lower income, having unemployed parents, being the eldest in the family, and having other family members with the same illness had higher mean scores for DAS compared with the other participants, though it did not reach statistical significance. The average depression score based on parents’ work was statistically significant (p = 0.024) if both parents are not working. The average anxiety score based on education level was also statistically significant (p = 0.020) and was higher in less educated patients. There were no other statistically significant differences for the other variables’ average scores.

**Table 3:** Comparison of depression, anxiety, and stress in the sample case study based on demographic variables. *p < 0.1

Demographic profile		Stress		Anxiety		Depression	
		Mean ± SD	p-Value	Mean ± SD	p-Value	Mean ± SD	p-Value
Age	14-18 years	4.47±3.63	0.033*	3.59±3.26	0.297	4.24±4.70	0.151
	≥19 years	7.09±4.41		4.67±3.72		5.91±3.79	
Gender	Male	6.81±3.84	0.435	4.16±3.64	0.651	5.71±3.86	0.623
	Female	5.94±4.82		4.58±3.61		5.19±4.35	
Marital status	Single	6.19±4.47	0.465	4.35±3.6	0.903	5.38±4.26	0.771
	Married	7.30±3.65		4.50±3.98		5.80±3.23	
Education level	Primary	6.92±4.70	0.939	7.08±2.88	0.020*	7.58±3.63	0.238
	Intermediate	6.00±4.36		3.13±2.64		4.67±4.34	
	High school	6.11±4.82		3.58±3.24		4.84±4.13	
	University	6.62±4.52		4.44±4.41		5.31±3.95	
Income	Low	7.55±4.76	0.511	5.64±5.22	0.361	4.27±3.29	
	Meddle	5.46±4.10		3.54±2.99		3.38±4.23	
	High	6.34±4.35		4.29±3.23		6.16±4.18	
Parents work	Work	5.80±3.73	0.40	3.88±3.48	0.382	4.04±3.22	0.024*
	Does not work	6.76±4.72		4.70±3.70		6.41±4.37	
Family members	Equal or less than 5 members	6.40±4.30	0.933	4.56±3.63	0.516	6.02±4.20	0.073
	More than 5 members	6.29±4.61		3.88±3.60		3.94±3.44	
Sequence in family	Oldest	7.73±4.98	0.154	5.33±3.66	0.488	5.73±3.69	0.259
	Middle	6.38±4		4.00±3.53		5.86±4.48	
	Youngest	4.30±4.22		4.30±3.92		3.50±2.59	
Affected family member	Yes	6.66±4.47	0.419	4.39±3.56	0.959	5.93±4.08	0.150
	No	5.67±4.07		4.33±3.82		4.28±3.95	

Table 4 shows the results of the independent samples t-test analysis and one-way ANOVA of DAS and factors affecting patients’ quality of life and patients’ thalassemia status and prognosis. Participants who were treated at MMCH had serum ferritin levels of more than 2,000 ng/mL, had a history of using parenteral iron chelator, had blood transfusion every three weeks, and showed higher mean scores for DAS compared with the other patients. Moreover, participants with a history of splenectomy had higher mean scores for anxiety and stress compared with those who did not undergo splenectomy. Participants who were satisfied with their medical care showed higher mean scores for DAS compared with the very satisfied and non-satisfied groups. The results showed that the stress scores had statistically significant differences between the medical care satisfaction groups after the ANOVA test was run (p = 0.021). Otherwise, there were no statistically significant differences for the other variables’ average scores.

**Table 4:** Comparison of depression, anxiety, and stress in the sample case study based on the factors affecting the quality of life and thalassemia prognosis. *p < 0.1

Quality of life and complications of the case variable		Stress		Anxiety		Depression	
		Mean ± SD	p-Value	Mean ± SD	p-Value	Mean ± SD	p-Value
Treating hospital	King Fahad Hospital	6.43±4.42	0.80	4.32±3.64	0.639	5.24±3.93	0.067
	Madinah Maternity and Children Hospital	7.00±3.00		5.33±3.22		9.67±5.77	
Medical care satisfaction	Very satisfied	5.32±4.01	0.021*	3.95±3.37	0.124	4.63±4.00	0.089
	Satisfied	8.63±4.57		5.63±4.00		7.11±3.78	
	Not satisfied	6.00±4.35		2.00±1.73		6.00±5.29	
Serum ferritin	Less than 2,000 ng/mL	5.67±4.92	0.476	5.13±3.74	0.351	6.33±4.81	0.341
	More than 2,000 ng/mL	6.60±4.18		4.13±3.75		5.17±3.85	
Blood transfusion frequency	Every 3 weeks	6.82±4.60	0.251	4.62±3.85	0.514	5.64±4.25	0.463
	Every 4 weeks	4.60±2.50		3.50±2.22		5.20±3.46	
	Every 5 weeks	6.37±4.35		2.50±2.12		2.00±0.00	
Hemoglobin before transfusion	Less than 9 gm/dL	6.18±4.59	0.515	4.33±3.71	0.852	5.49±3.86	0.888
	More than 9 gm/dL	7.08±3.33		4.54±3.31		5.31±5.04	
History of splenectomy	Yes	7.25±4.09	0.060	4.64±3.74	0.496	5.81±3.31	0.427
	No	5.15±4.47		4.00±3.44		5.99±4.99	
History of using deferoxamine as iron chelator	Yes	6.69±4.40	0.406	4.64±3.65	0.394	6.07±4.04	0.084
	No	5.70±4.27		3.80±3.53		4.15±3.98	
Satisfaction from family psychological support	Satisfied	6.25±4.52	0.621	4.31±3.62	0.755	5.21±4.13	0.295
	Not satisfied	7.00±3.43		4.70±3.68		6.70±3.80	
Satisfaction from medical team psychological support	Satisfied	6.04±4.06	0.144	4.57±3.48	0.305	5.62±4.14	0.429
	Not satisfied	8.33±5.63		3.22±4.30		4.44±3.84	

In terms of satisfaction from the psychological support of the participants’ families and their medical team, the participants who were satisfied with their families’ and medical team’s psychological support showed lower mean scores for DAS compared with the participants who were not satisfied. Independent samples t-test showed that there were no statistically significant differences in the means of DAS and satisfaction with the psychological support of the participants’ families and medical team.

## Discussion

In our study, depression symptoms were found in 60% of patients, and particularly the moderate and severe depression symptoms were reported in 40% of our patients, which is considered among the highest prevalence reported internationally [9-11]. In our analysis, it is clear that patients with BTM had very high anxiety and stress scores, which are comparable to international result, as stress was detected in up to 38.7% of our patients, being severe in almost 10% and moderate in 16% of them. Anxiety on the other hand was detected in half of the studied group, where 23% had severe anxiety and 6.5% were within the moderate range of anxiety.

Several studies reported the prevalence of depression in thalassemia patients internationally, ranging from 10% to 50%, but we could not find any publication from Saudi Arabia [7,8,13]. Different reports from Iran reported that depression in BTM patients is a predominant presentation ranging between 12% and 30% and that up to 54% of patients found to have a type of psychopathological disorder [14-17]. More serious psychological complications in BTM patients can present, as it was reported in a study conducted in Iran that up to 27.3% of patients considered suicide in the year before the study [18].

In Arab communities, in 2012, a study reported similar findings from 80 patients in Lebanon where 35% of patients were depressed [19]. In the study by Al-Hakeim et al., it was reported that major depression in children with BTM was strongly associated with the number of blood transfusions, iron overload, and increased levels of interleukin-1ß [5].

In Saudi Arabia, a study conducted on patients with sickle cell anemia from the Qatief area reported that 48% of patients had symptoms of depression. This may suggest that all patients with hereditary hemoglobinopathy or other chronic medical diagnoses are at risk of psychological disorders [20].

Adolescents with chronic medical diagnoses such as BTM are at higher risk of developing psychological disorders such as having identity issues and hormonal changes, but children and older patients are still at risk of developing serious psychological and psychiatric disorders related to the stress they are facing because of the disease [13]. In our study, this was clear as older patients reported higher depression and anxiety rates compared to younger patients who reported more stress, though it did not reach a statistical significance. It is well reported that life expectancy of BTM patients had improved dramatically with the improvement of medical care, but this was associated with higher rate of depression as patients had lower school performance, less job opportunities, less marriage chances, less independency, and being socially isolated [8]. In our study, patients aged less than 18 years had higher stress (statistically significant|), whereas patients older than 18 years had more depression and anxiety, but not reaching a significant statistical difference, and married participants obtained higher mean scores for DAS than single participants. Participants with a lower income, with unemployed parents, being the eldest in the family, and having other family members with the same illness had higher mean scores for DAS compared with the other participants, though it did not reach statistical significance.

Assessment of quality of life in thalassemia patients was discussed locally and internationally and it was assessed in Almadinah Almunawwarah and Jeddah, which included emotional functional assessment in addition to physical and social functions, with results suggesting an effect on all domains compared to normal population [21-23]; these results support our findings in this study.

It seems that the stress in thalassemia patients can also affect the care givers of those patients, who were also found to have symptoms of depression (around 30% of them) [24].

Social and financial support should be encouraged as most of our patients were severely financially affected or in need. Specialized charity societies can provide adequate support like the one in Almadinah Almunawwarah, namely Madinah Hereditary Charity Society. This was evident in our results as the participants who were satisfied with their families’ and medical team’s psychological support showed lower mean scores for DAS compared with the participants who were not satisfied, as poor family adjustment was also documented in a multicultural study, and this is expected to have a significant effect on the psychological well-being of patients [15,25]. This was reflected in our results as the average depression score based on parents’ work was statistically significant (p = 0.024) if parents are not working. The difference in the average anxiety score based on education level was also statistically significant (p = 0.020) and was higher in less educated patients.

Oral chelators such as deferiprone and deferasirox had improved the compliance of patients to take their medication regularly, but unfortunately it is not available for all patients and deferoxamine is still being used widely by our patients as injections subcutaneously using a pump for eight hours a day and for five days a week [26]. Because of iron overload, patients may have complications in different organs, including endocrinopathies, heart failure, conduction abnormalities, liver derangement, and splenomegaly, which requires splenectomy [9]. Recent data suggested a possible link between neurotransmitter abnormalities in thalassemia patients and the occurrence of depression [27-30]. Our results showed higher scores in all three domains in patients’ receiving deferoxamine compared to oral chelators, but the difference did not reach a statistical significance. Our results showed that the stress scores had statistically significant differences between the medical care satisfaction groups. Otherwise, there were no statistically significant differences for the other variables’ average scores.

A limitation of this study is that the study has a relatively small number of patients enrolled, with no local studies to compare our results with. A proper clinical psychological evaluation will strengthen our findings as the self-assessment of DAS in our study was conducted through telephone.

## Conclusions

Patients with BTM are at risk of developing psychological consequences, and this might affect their course of the disease and both short- and long-term sequelae. Early diagnosis and proper support and management are highly needed to help patients coping with their disease and improve their quality of life.

Referral of all screened patients with symptoms of depression, anxiety, or stress to a psychiatry clinic for proper intervention is recommended. Further studies with a long follow-up plan and a multicenter national enrolment are highly needed for optimizing the right care of those patients.
